# Characteristics of Physiological Parameters of Japanese Black Calves Relate to Carcass Weight

**DOI:** 10.3390/ani13030487

**Published:** 2023-01-31

**Authors:** Shotaro Arakawa, Minji Kim, Tatsuya Aonuma, Michihiro Takagi, Satoshi Watanabe, Huseong Lee, Koki Nishihara, Satoshi Haga, Yoshinobu Uemoto, Sanggun Roh

**Affiliations:** 1 Graduate School of Agricultural Science, Tohoku University, Sendai 980-8572, Japan; 2 Miyagi Prefectural Animal Industry Experiment Station, Oosaki 989-6445, Japan

**Keywords:** Japanese Black cattle, metabolic profile test, carcass weight, growth performance

## Abstract

**Simple Summary:**

Withers height and chest girth in calves were significantly correlated with carcass weight and body weight, regardless of sex. Meanwhile, the relationship between blood metabolites and carcass weight differed according to sex. Body measurements and blood metabolites measured during the growing period could be used to determine the nutritional and physiological status of cattle and predict the final carcass weight, but the animal factor, such as sex, should be considered.

**Abstract:**

This study aimed to identify the growth performance and blood factors associated with carcass weight in Japanese Black calves based on 675 performance tests and field carcass records. We measured the body weight, withers height, and chest girth at the start of fattening age (approximately 8–10 months) and analyzed eight blood factors, including vitamins and metabolites. Single- and two-trait animal models were used to estimate the heritability and genetic correlations. The heritability estimates for growth performance were moderate to high (ranging from 0.48 to 0.74), and those for blood metabolites were low to moderate (ranging from 0.19 to 0.51). Estimates for genetic correlations of carcass or body weight with body weight, withers height, and chest girth were high (ranging from 0.42 to 0.80). The body weight and withers height at 8 months of age are possibly closely related to the final carcass weight. The blood metabolites associated with body weight were vitamin E in steers (castrated males) and β-carotene in heifers. Our findings indicate that body measurements and blood metabolites measured during the growing period could be used to determine the nutritional and physiological status of cattle as well as predict carcass weight.

## 1. Introduction

Metabolites, substrates, or products of metabolic processes are involved in many biological functions such as energy metabolism, signaling, stimulatory and inhibitory effects on enzymes, and immunological defense. The metabolic profile test (MPT) is a clinical method for analyzing and interpreting various metabolites with physiological functions and has been widely used to understand the nutritional status and metabolic activity of animals [1,2,3,4]. The MPT is based on the notion that the nutritional and metabolic status of animals is reflected in certain blood metabolites [3] and that the metabolite concentrations depend on various biotic and abiotic conditions [5,6,7,8]. Therefore, to apply MPT for livestock feeding management, standard values and features of blood metabolites established under various environmental and feeding conditions are required.

The physiological features of Japanese Black cattle, such as blood metabolites, possibly differ from those of other cattle breeds owing to differences in their genetic background, rearing environment, and feeding management [9,10]. Japanese Black cattle have improved through the years to produce high-quality beef using a unique feeding management system [11,12]. To induce a considerable accumulation of intramuscular fat, farms raising Japanese Black cattle have been used as feeding systems focusing on fattening cattle at 11–30 months of age [13,14]. During the fattening period, Japanese Black cattle are fed a high-energy diet with low levels of roughage until slaughter at 28–30 months of age [13,14]. Therefore, the feeding management of growing Japanese Black cattle aims to promote rumen development and improve adaptation to long-term fattening. The feeding management during the weaning and growing periods may affect the growth performance and carcass traits of beef cattle. Fluharty et al. [15] reported that Angus crossbred calves offered concentrate diets ad libitum showed improved growth rates compared to calves for programmed intake. They also reported that early weaning with high-concentrate diets accelerated the growth rate and fat deposition in calves. A study on Angus × Simmental cattle [16] suggested that different dietary sources and energy intake during the growing period affected the growth curve and amount of intramuscular fat deposition. In particular, the calves fed starch-based diets have higher marbling scores. However, because the current feeding management of Japanese Black cattle for producing high-quality beef mainly focuses on the fattening period, studies on the physiological characteristics related to productivity during the weaning and growing periods are lacking.

Therefore, this study aimed to identify the growth performance and blood factors associated with carcass weight (CW) in Japanese Black calves. Growth performance and various blood metabolites were measured at the start of fattening age (approximately 8–10 months) to examine their potential as productivity indicators in Japanese Black calves.

## 2. Materials and Methods

### 2.1. Animals and Data Collection

All experimental animals were managed according to the Guidelines for the Care and Use of Laboratory Animals established by the National Livestock Breeding Center (NLBC). Japanese Black calves were fed until slaughter in 19 farms in Miyagi Prefecture between 2008 and 2017 for on-farm progeny testing. The feeding program in each farm was conducted with the adequacy rate of the diet based on the nutrient requirements of the Japanese Feeding Standard for Beef cattle [17]. Water was always freely available, and management was conducted in accordance with farm practices. For growth performance, body weight (BW), withers height (WH), and chest girth (CG) at the start of fattening (approximately 8–10 months) were measured from July to November each year. These three traits are regarded as growth performance traits. All cattle were slaughtered at approximately 25–34 months of age, and CW was evaluated by official graders in accordance with the Japanese Meat Grading Association [18].

A total of 704 Japanese Black cattle (steer N = 398 and heifer N = 306) with records of growth performance and CW were obtained and used to measure blood metabolite levels. For each trait, animals with phenotypic values exceeding the mean ± 4 SD were excluded after measuring the blood metabolites, and only animals with phenotypic values for all traits were extracted. A total of 675 animals (steer N = 382 and heifer N = 293) were included in the statistical analyses. The descriptive statistics of 675 Japanese Black cattle for growth performance, blood metabolites, and CW are shown in Table 1. In addition, no statistical differences between farms were observed in all statistics.

### 2.2. Blood Sampling and Blood Metabolites Profiling

Blood samples were collected during growth performance measurements, and each blood metabolite concentration was measured. Blood samples were collected from the jugular vein at 10:00 into serum-separating tubes (VP-AS109K, Tokyo Garasu Kikai Co., Ltd. Tokyo, Japan). All samples were transferred to the laboratory on ice and stored until centrifugation at 1500× *g* for 15 min. Plasma samples were stored at −30 °C until metabolic profiling. Vitamin A (VitA), vitamin E (VitE), and β-carotene (bC) levels were measured using high-performance liquid chromatography (HPLC) [19]. Total cholesterol (TC), albumin (ALB), blood urea nitrogen (BUN), aspartate aminotransferase (AST), and gamma-glutamyl transferase (GGT) levels were measured by the colorimetric method using an Automatic Biochemical Analyzer (DRI-CHEM NV700, Fuji Film Co., Tokyo, Japan). These eight traits are regarded as blood metabolite traits.

### 2.3. Genetic Analyses

To estimate (co)variance components with standard errors (SEs), we used ASReml 4.1 software [20] using the following animal model:

(1) $$y_{ijkl}=\mu_{i}+sex_{ij}+test_{ik}+b_{1}x_{il}+u_{il}+e_{ijkl}$$

where $y_{ijkl}$ is the observation of animal *l* for trait *i*; $\mu_{i}$ is the total mean for trait *i*; $sex_{ij}$ is the fixed effect of sex *j* (two levels) for trait *i*; $test_{ik}$ is the fixed effect of on-farm progeny test group *k* (20 levels) for trait *i*; $b_{1}x_{il}$ is the linear regression coefficient ($b_{1}$) on month of age ($x_{il}$) of animal *l* for trait *i*; $x_{il}$ is the fattening starting age for growth performance and blood metabolites and the slaughter age for CW; $u_{il}$ is the random additive genetic effect of animal *l* for trait *i*; $e_{ijkl}$ is the error effect. The pedigrees were traced back to five generations, and 4455 animals were used for pedigree information. A single-trait animal model for estimating heritability and a two-trait animal model for estimating genetic correlations were applied using model (1).

### 2.4. Non-Genetic Analyses

To characterize the factors affecting CW, non-genetic analyses were performed using the R software v4.0.1 accessed on 6 June 2020 (http://www.r-project.org). To account for the differences in age at phenotyping among animals, a total of 12 traits, including 3 growth performance traits, 8 blood metabolites, and CW, were first adjusted by fitting the linear model with the *glm* function as follows:

$$y_{ij}=\mu +test_{i}+b_{1}x_{j}+e_{ij},$$

where $y_{ij}$ is the observation of animal *j*; μ is the total mean; $test_{i}$ is the fixed effect of on-farm progeny test group *i* (20 levels); $b_{1}x_{j}$ is the linear regression coefficient ($b_{1}$) on month of age ($x_{j}$) of animal *j*; $x_{j}$ is the fattening starting age for growth performance and blood metabolites and the slaughter age for CW; $e_{ij}$ is the error effect. The adjusted phenotypic value ($adj\_y_{ij}$) was then derived as follows:

$$adj\_y_{ij}=\hat{\mu }+{\hat{b}}_{1}x+{\hat{e}}_{ij},$$

where the specific months of age for the fattening starting age (8 months) and slaughter age (29 months) were assigned to *x* in the model.

Adjusted phenotypic values were used to calculate phenotypic correlations among traits and to investigate the impact of physiological differences in growth performance and blood metabolites on CW. The Japanese Black cattle in the high-CW group (steer N = 64 and heifer N = 48) and low-CW group (steer N = 60 and heifer N = 48) were selected from the higher (above mean + SD) and lower (below mean − SD) tails of the adjusted CW phenotypic distribution in each sex in the population, respectively. The differences in growth performance, blood metabolites, and CW were tested between the high- and low-CW groups using Student’s *t*-test. The present study considered differences statistically significant at *p* < 0.05. We also evaluated the impact of physiological differences in growth performance and blood metabolites on BW using the same method described above. The high-BW group (steer, N = 57; heifer, N = 43) and low-BW group (steer, N = 55; heifer, N = 46) were selected, and the differences were tested.

To identify the parameters related to CW using adjusted phenotypic values, regression tree analysis was performed using the *ctree* function in the R package partykit [21]. The regression tree estimates the relationship between dependent and independent variables through binary recursive split zones in a conditional inference framework [21]. CW was used as the dependent variable, and sex, three growth performance traits, and eight blood metabolites were used as independent variables. For significance testing, a Bonferroni-adjusted *p*-value of *p* < 0.05 was used to assess all splits to avoid overfitting and biased selection among variables. We also investigated the parameters related to BW using the same method described above. BW was used as the dependent variable, and sex and eight blood metabolites were used as independent variables.

## 3. Results and Discussion

We evaluated growth performance and blood metabolites in calves before the fattening period associated with carcass traits in slaughtered Japanese Black cattle. Our findings suggest that some factors, such as blood metabolites and growth performance, obtained from calves might be associated with carcass weight, and the relationship between these factors and carcass traits may be affected by sex. The major issues are discussed as follows.

### 3.1. Genetic Relationships among Growth Performance, Blood Metabolites, and Carcass Traits in Japanese Black Calves

Genetic parameter estimations and correlations for growth performance, blood metabolite levels, and carcass weight are shown in Table 2. The heritability estimates for BW, WH, and CG were 0.48, 0.51, and 0.74, respectively, and those for blood metabolite estimates were low to moderate (ranging from 0.19 to 0.51). The heritability estimates of CW were moderate (0.46). In particular, our estimates of the heritability of CG appeared to be high.

Mukai et al. [22] reported that heritability estimates were low to moderate for BW, WH, and CG (ranging from 0.19 to 0.36) in Japanese Black calves at 8–11 months of age. Okanishi et al. [23] have reported heritability estimates of 0.39, 0.36, and 0.36 for BW, WH, and CG, respectively, in Japanese Black bull calves. Shinoda et al. [24] reported heritability estimates of 0.34 for CW in Japanese Shorthorn cattle. Compared to the above estimates, our heritability estimates of growth traits were slightly higher than those of literal values; in particular, the estimate was notably higher for CG. The differences in heritability estimates may have occurred due to the animal and environmental variances used in performance tests. The heritability estimates of the blood metabolites were low to moderate. Although there are a few studies on heritability estimates of serum VitA in beef cattle, some previous studies have reported that the genetic value of vitamins differs depending on the sex and age of the cattle. Taylor et al. [25] reported the heritability estimates of serum VitA concentrations in Hereford cattle and showed estimates of 0.19 and 0.17 for males at weaning and 600 days of age, respectively, and 0.16, 0.13, and 0.04 for females at weaning, 340 days of age, and 710 days of age, respectively. More recently, Kato et al. [26] reported that heritability estimates for serum VitA significantly decreased from 0.37 to 0.07 as the stages progressed in an F1 cross between Japanese Black sires and Holstein dams. These results suggest that the genetic influence on serum VitA concentrations is moderate when cattle are young but is relatively low in later periods of fattening. The reason for this is unclear, but it is possibly related to feeding management with VitA control. A previous study reported that TC estimates were moderate to high (approximately 0.35 to 0.64) throughout the stages in crossbred Black cattle [26], similar to that observed in our study. Additionally, Sasaki et al. [27] reported that the heritability estimates of TC showed estimates of 0.28, 0.61, and 0.83 for 10 to 30, 40 to 60, and 70 to 90 days of age in Holstein calves, respectively. The genetic estimates of TC showed moderate to high heritability across breeds and ages. Genetic differences may be a factor of direct variation in metabolome profiles, or an indirect factor through genetic influences on physiology, behavior, and feeding management. Our findings were investigated under the similar feeding protocol (although environmental factors are not exactly the same), thus the high heritability of TC seems to be due to genetic differences depending on parents than indirect factors. Therefore, we could suggest that TC concentrations would be heritable traits in Japanese Black cattle.

The genetic correlation estimates for growth performance and blood metabolite levels in CW and BW are shown in Table 2. The genetic correlation estimates of CW with BW, WH, and CG were high (0.55, 0.61, and 0.42, respectively). In addition, the genetic correlation estimates of BW with WH and CG were high (0.71 and 0.80, respectively). Our results indicate that the growth performance measured during the growing period positively correlates with BW and CW. Mukai et al. [22] reported that CW was highly genetically correlated with WH (ranging from 0.72 to 0.75) and CG (ranging from 0.64 to 0.79) in Japanese Black bulls during the growing period. Baco et al. [28] reported that the BW of Japanese Black cows has a positive genetic correlation with WH (r = 0.63) and CG (r = 0.86) and that there is a moderate to high correlation between each body measurement. Therefore, our findings and those of previous studies suggest that cattle with high WH and CG may be superior in growth and CW, and that body measurements during the growing period might be used to predict the CW of Japanese Black cattle. The genetic correlation estimates of CW with VitA, bC, and BUN were −0.38, −0.39, and −0.40, respectively. The genetic correlation estimates of BW and blood metabolites were moderate to high (ranging from −0.30 to −0.69), except for bC (0.01). In the present study, certain blood metabolites, such as VitA and BUN, were negatively correlated with BW and CW; our findings suggest that genetic selection based on these metabolites might be useful in selecting the cattle with higher BW and CW. Physiologically, VitA and BUN are associated with various physiological activities and protein metabolisms, indicating that the metabolic states at the start of fattening affect BW and CW; however, the blood metabolites had a relatively low genetic correlation with BW and CW compared with that of growth performance. One of the reasons for this might be that blood metabolites have low heritability estimates compared to that of growth performance.

**Figure 1:**
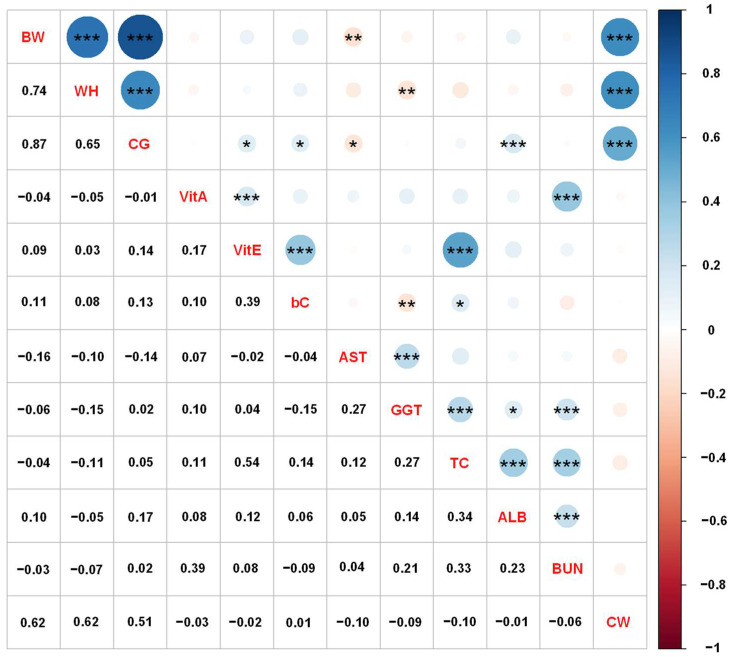
Phenotypic correlation coefficients among the growth performance, blood metabolites, and carcass weight (CW) in Japanese Black cattle. The correlation coefficients are shown on the lower diagonal, and the blue and red colors of the circles on the upper diagonal indicate positive and negative correlations, respectively. Significances of correlation coefficients are shown as asterisks accordingly *: *p* < 0.05, **: *p* < 0.01, and ***: *p* < 0.001 on the upper diagonal. Abbreviations of traits are shown in Table 1.

Figure 1 shows the phenotypic correlations of growth performance, blood metabolite levels, and carcass traits in Japanese Black cattle using adjusted phenotypic values. The phenotypic correlation among the growth performance was positively high (ranging from 0.65 to 0.87), and the correlations among the blood metabolites were low to moderate (ranging from −0.15 to 0.54). Phenotype correlations between growth performance and blood metabolites were low (ranging from −0.16 to 0.17). The correlations of CW with BW, WH, and CG were 0.62, 0.62, and 0.51, respectively, with those between CW and blood metabolites being low (ranging from −0.10 to 0.01). In contrast, the genetic correlation estimates of CW with VitA, bC, and BUN showed negative and moderate values, suggesting that the correlation was significantly affected by genetic factors.

Our results indicated that the phenotype correlations among the growth performance and blood metabolites collected from growing Japanese Black cattle were similar to the genetic correlation estimates. CG and WH had high correlations with CW and BW, and the correlation between CG and WH was also high. As reported above, the genetic correlations of CG and WH with CW and BW were estimated to be moderate to high (ranging from 0.42 to 0.80; Table 2). Phenotypic correlations were relatively high compared with genetic correlations. Regarding the heritability estimates, genetic and phenotypic correlations for body measurements, Baco et al. [28] reported that phenotypic correlation of body weight in Japanese Black cattle have positive correlations with CG (r = 0.57) and WH (r = 0.72), and the estimates between CG and WH were 0.40.

In summary, growth performance, such as CG and WH, measured during the growing period could be used as an indicator to predict the final CW in Japanese Black steers and heifers. However, blood metabolites had a lower phenotypic correlation with parameters other than growth performance. Therefore, it is necessary to thoroughly investigate the relationship between blood metabolites, growth performance, and carcass traits in various animal factors, such as breed and sex, to predict the productivity of Japanese Black cattle.

### 3.2. Characteristics of Growth Performance and Blood Metabolites According to CW

Table 3 shows the comparison of growth performance and blood metabolites in high and low groups based on the CW. Significant differences were observed in BW, WH, and CW for both sexes. This indicates that growth traits during the growing period positively correlate with CW among the final fattening performance.

According to a comparative study of body measurements in different breeds [29], the correlations between live weight and body measurements were moderate to strongly positive (r = 0.40–0.83). Additionally, the WH and haunch width strongly correlated with live BW regardless of cattle bred. The blood GGT and BUN levels differed according to the CW group (high vs. low) in steers. BUN is an end product of protein metabolism and is a sensitive indicator of the balance between the amount and availability of crude protein and energy fed to ruminants [30]. Kim et al. [11] investigated the correlations between blood metabolites and carcass traits in Japanese Black steers during the early and late fattening periods (13 and 28 months of age), and blood BUN concentrations were positively correlated with growth rate and CW in the late fattening period (r = 0.429 and 0.310, respectively). BUN concentrations increase with high-protein diets owing to increased ammonia production [31]. As such, several studies [12,31] reported that serum BUN levels positively correlate with feed intake and carcass traits in beef cattle. We expected that BUN levels are positively correlated with CW, but the findings revealed that the cattle with low BUN levels at the start of fattening had a relatively high CW. Regarding an unexpected result, we speculated that the difference in feed intake at the start of fattening affected the final CW and at the same time made a difference in the BUN levels. Therefore, the BUN level could be used as an indirect indicator of CW. In general, the Japanese Feeding Standard for beef cattle recommends that during this period, roughage should comprise ≥40% of the total feed ration and limit the intake of high-grain diets to promote growth and prevent excessive fattening. Our results showed that blood GGT levels were significantly lower in steers with high CW. Blood GGT is mainly released from the liver and bile duct into the bloodstream when the hepatocytes are damaged by stimulation, such as long-term high-energy feeding, metabolic disease, and mold toxins [30,32]. Previous studies on Japanese Black steers [33,34] have shown that blood metabolites related to liver function tend to increase during the late fattening phases. Therefore, the cattle with low GGT levels at the start of fattening are likely to have a normal liver function and lower metabolic load, leading to improved productivity, such as CW. The blood bC levels differed according to the CW group (high vs. low) in heifers. bC, a pro-vitamin A carotenoid, has been studied in relation to reproductive performance because it improves immune function and has antioxidant properties [35,36,37]. Aragona et al. [38] reported that supplementing bC to Holstein cows in the prepartum period can improve the blood bC levels and feed efficiency of calves. In addition, bC might be related to rumen microbial growth. Yan et al. [39] reported that incubation of bC in goat rumen fluid resulted in lesser NH_3_-N levels and greater microbial crude protein levels than those of the control group with no bC. This result suggests that bC can promote microbial growth by increasing NH_3_-N utilization. Our findings are consistent with previous studies [38,39] that bC promotes feed efficiency and rumen microbial growth related to the growth of ruminants. However, the blood levels of bC in steer were not significant according to the CW group. The bC metabolism is possibly affected by animal factors, such as sex and age [40,41]. Previous studies on the relationship between blood metabolites and carcass traits have mainly focused on the fattening period after 12 months of age in bulls or steers; thus, there is a lack of data measured for the various animal factors, such as feeding period, breed, and sex. Therefore, it is necessary to investigate the relationship between blood metabolites and carcass traits in various animal factors.

Table 4 shows the comparison of growth performance and blood metabolites in high and low groups based on the BW. Significant differences were observed in WH and CG for both sexes.

Ozkaya and Bozkurt [42] reported that WH and CG were positively correlated with BW (ranging from 0.66 to 0.95) in Holstein, Brown Swiss, and crossbred cattle. There were no significant differences in blood concentrations of VitA, GGT, TC, and BUN between the high and low groups, whereas the bC levels differed significantly between both sexes. As mentioned above, bC promotes feed efficiency [38] and rumen microbial growth [39] related to the growth of ruminants. Accordingly, increased bC concentrations of the high-BW group apparently are related physiological activation of bC. Blood VitE levels were significantly higher in the high-BW group than in the low-BW group in steers, and there was a similar trend in heifers (*p* < 0.1). VitE, a lipid-soluble antioxidant, has been studied in relation to reproductive performance [43], the health of the transition period [44], and meat quality [45] in cattle because it improves immune function and has antioxidant properties. Various stressors, such as weaning, transportation, and changing feed management, stimulate an acute inflammatory response [46] and increase oxidative stress [47], resulting in a negative effect on the health and performance of cattle [48]. Therefore, VitE can reduce oxidative stress associated with feed management and affect the growth and productivity of cattle. In the present study, the increased VitE levels in the high-BW group were probably related to indirect growth promotion due to the antioxidant function of VitE, although it was not significant in comparison with the CW group. Blood AST levels were significantly higher in the low-BW group in steers. AST, an indicator of liver function with GGT, increased in low-BW steers. However, it is considered that indicator specificity for liver function is weak compared to GGT because AST is secreted from various tissues, including liver tissues. In heifer calves, blood ALB levels differed according to BW (high vs. low). Blood ALB level is a sensitive nutritional indicator associated with protein intake and reflects a relatively short-term nutritional status [49]. Additionally, the ALB concentration can be used as an indicator to indirectly predict liver function and protein metabolism in cattle [50]. In the present study, blood ALB concentrations were similar to those reported previously [51]. Therefore, the increased ALB levels in the high-BW group are possibly related to differences in the concentrate and protein intake between the high- and low-BW groups rather than to abnormal liver function. The growth performance and blood parameters significantly differed between the high- and low-BW groups in Japanese Black cattle. Furthermore, comparing blood metabolites, the relationship between blood parameters and BW showed a similar trend in steers and heifers, but some parameters indicated significant differences between steers and heifers. Therefore, we performed a regression tree analysis to identify the parameters related to CW and BW using the adjusted phenotypic values.

### 3.3. Regression Tree for CW and BW in Japanese Black Calves

**Figure 2:**
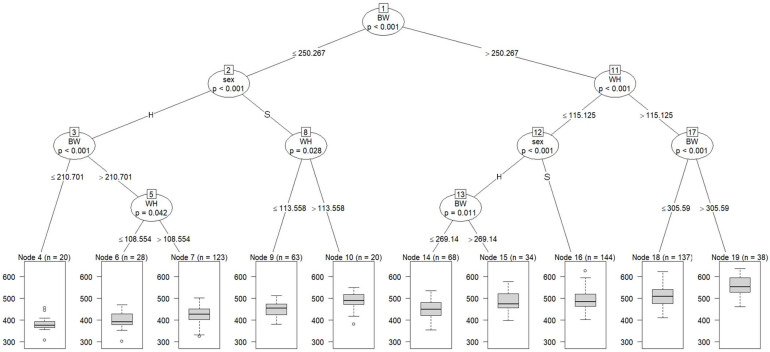
The graphical model of regression tree for carcass weight. A total of 675 animals with the adjusted phenotypic values for the fattening starting age (8 months) and the slaughter age (29 months) were used in the analysis. Carcass weight was used as a dependent variable, and sex (S = steer, H = heifer), 3 growth performance traits, and 8 blood metabolites were used as independent variables. A total of 675 animals with the adjusted phenotypic values for the fattening starting age (8 months) and the slaughter age (29 months) were used in the analysis. Abbreviations of traits are shown in Table 1.

Figure 2 shows a regression tree with CW adjusted at 29 months of age as the dependent variable and blood metabolites and growth performance adjusted at 8 months of age and sex as independent variables. All cattle were split into subgroups: Node 2 (a subgroup of cattle with BW ≤ 250.267 Kg) and Node 11 (a subgroup of cattle with BW > 250.267 Kg). At the second tree depth, Node 2 was partitioned into Nodes 3 and 8 according to sex, and Node 11 was partitioned into Node 12 (a subgroup of cattle with WH ≤ 115.125 cm) and 17 (a subgroup of cattle with WH > 115.125 cm). At the third depth, Node 3 was split into Node 4 (a subgroup of cattle with BW ≤ 210.701 Kg) and Node 5 (a subgroup of cattle with BW > 210.701 Kg); Node 8 was split into Node 9 (a subgroup of cattle with WH ≤ 113.558 cm) and Node 10 (a subgroup of cattle with WH > 113.558 cm); Node 12 was split into Node 13 and Node 16 according to sex; and Node 17 was partitioned into Node 18 (a subgroup of cattle with BW ≤ 305.59 Kg) and Node 19 (a subgroup of cattle with BW > 305.59 Kg). At the fourth depth, Node 13 was divided into Nodes 14 and 15, and Node 5 was divided into Nodes 6 and 7, according to WH.

Our findings suggest that BW at 8 months of age is possibly closely related to the final CW. WH appeared at the second and third tree depths on the left side of the figure, suggesting that WH is also closely related to CW. Cattle with high BW and WH at 8 months of age are thought to have an advantage in adapting to high-energy diets and maintaining productivity during the fattening period. During the period up to approximately 12 months of age, bones, intestines, digestive organs (especially the rumen), and muscles of calves grow intensely. Thus, the feeding management of growing Japanese Black calves aims to promote rumen development and improve adaptation to long-term fattening, and feeding high-quality roughage is recommended during this period [52]. Therefore, our findings suggest that BW and WH at 8 months of age might be used as productivity indicators and that feeding management during the growing period is a very important factor for the growth and productivity of Japanese Black cattle.

**Figure 3:**
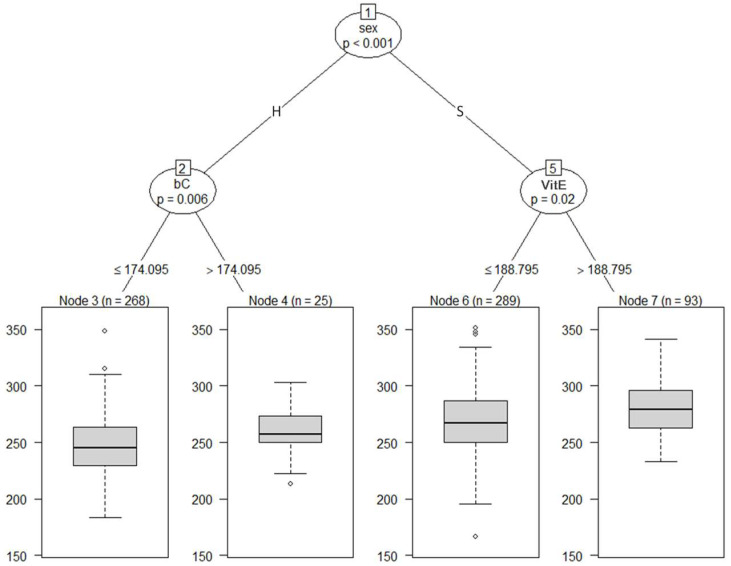
The graphical model of regression tree for body weight. A total of 675 animals with the adjusted phenotypic values for the fattening starting age (8 months) and the slaughter age (29 months) were used in the analysis. Body weight was used as a dependent variable, and sex (S = steer, H = heifer) and 8 blood metabolites were used as independent variables. A total of 675 animals with the adjusted phenotypic values for the fattening starting age (8 months) were used in the analysis. Abbreviations of traits are shown in Table 1.

Figure 3 shows the regression tree with BW adjusted at 8 months of age as the dependent variable and blood metabolites adjusted at 8 months of age and sex as independent variables. All cattle were split into subgroups, Nodes 2 and 5, according to sex. At the second depth, Node 2 was divided into Node 3 (a subgroup of the cattle with bC ≤ 174.095 µg/dL) and 4 (a subgroup of the cattle with bC > 174.095 µg/dL); and Node 5 was partitioned into Node 6 (a subgroup of the cattle with VitE ≤ 188.795 µg/dL) and 7 (a subgroup of the cattle with VitE > 188.795 µg/dL). After being separated by sex in the first branch, the second branch showed VitE for steers and bC for heifers. Our findings revealed that the blood metabolites associated with BW were VitE in steers and bC in heifers.

## 4. Conclusions

Our study aimed to identify the growth performance and blood metabolites associated with CW and BW in Japanese Black calves. The heritability estimates of CG were especially high, and those of blood metabolites were low to moderate in Japanese Black cattle. WH and CG were significantly correlated with CW, regardless of sex, although the relationship with blood metabolites differed according to sex. The regression tree showed that the blood metabolites associated with BW were VitE in steers and bC in heifers. Our findings suggest that the body measurements and blood metabolites measured during the growing period could be used to determine the nutritional and physiological status of Japanese Black calves bred and raised in accordance with the typical feeding systems in Japan and predict the final CW. Additionally, genetic selection based on the parameters identified from our study might be useful in selecting the cattle with higher BW and CW. However, the present study focused on blood metabolite concentrations at the beginning of the fattening period, and changes in blood metabolites of growing Japanese Black cattle over time remain unknown. Therefore, it is necessary to investigate the changes in blood metabolite levels during the entire period and identify factors related to fattening and productivity.

## Figures and Tables

**Table 1:** Descriptive statistics of 675 Japanese Black cattle for growth performance, blood metabolites, and carcass weight. Growth performance and blood metabolites were measured at the start of fattening (approximately 8–10 months). SD: Standard deviation.

Traits	Abbreviations	Min	Max	Mean	SD
**Growth performance**					
Body weight (Kg)	BW	164	397	280.95	34.12
Wither height (cm)	WH	103	127	115.07	3.72
Chest girth (cm)	CG	130	172	151.91	6.58
**Blood metabolites**					
Vitamin A (IU/dL)	VitA	39.07	213.79	105.04	28.78
Vitamin E (μg/dL)	VitE	27.98	518.02	172.3	78.75
β-carotene (μg/dL)	bC	2.98	282.62	69.2	52.49
Asparate aminotransferase (U/L)	AST	46	170	71.23	16.68
γ-glutamyl transferase (U/L)	GGT	12	56	22.69	5.22
Total cholesterol (mg/dL)	TC	52	279	132.12	39.64
Albumin (g/dL)	ALB	2.9	4.8	3.77	0.26
Blood urea nitrogen (mg/dL)	BUN	4.4	26.8	13.92	3.46
**Carcass weight (Kg)**	CW	317	670.5	482.24	65.01

**Table 2:** Genetic parameter estimations for growth performance, blood metabolites, and carcass weight (CW) in Japanese Black cattle. Growth performance and blood metabolites were measured at the start of fattening (approximately 8–10 months). Genetic (r_G_) correlation estimates of growth performance and blood metabolites in CW and body weight (BW). SE: Standard error. Abbreviations of traits are shown in Table 1.

Traits	Genetic Variance		Heritability		CW		BW	
	Estimate	SE	Estimate	SE	r_G_	SE	r_G_	SE
**Growth performance**								
BW	388.25	155.92	0.48	0.17	0.55	0.20	-	-
WH	5.29	2.08	0.51	0.17	0.61	0.19	0.71	0.15
CG	21.92	7.14	0.74	0.19	0.42	0.22	0.80	0.09
**Blood metabolites**								
VitA	185.31	94.54	0.30	0.14	−0.38	0.27	−0.66	0.19
VitE	2115.56	983.98	0.36	0.15	0.00	0.31	−0.30	0.29
bC	447.86	290.82	0.19	0.12	−0.39	0.38	0.01	0.37
AST	106.55	50.74	0.39	0.16	−0.08	0.31	−0.41	0.27
GGT	10.08	4.54	0.38	0.15	−0.08	0.30	−0.57	0.25
TC	659.16	282.90	0.47	0.17	−0.15	0.29	−0.69	0.23
ALB	0.01	0.01	0.25	0.14	0.04	0.35	−0.37	0.33
BUN	6.39	2.53	0.51	0.17	−0.40	0.24	−0.64	0.18
**CW**	1287.57	523.40	0.46	0.16	-	-	-	-

**Table 3:** Comparisons of growth performance, blood metabolites, and carcass weight between high and low carcass weight (CW) groups. Growth performance and blood metabolites were measured at the start of fattening (approximately 8–10 months). SE: Standard error. Abbreviations of traits are shown in Table 1.

Traits	Steer					Heifer				
	High (N = 64)		Low (N = 60)		*p*-Value	High (N = 48)		Low (N = 48)		*p*-Value
	Mean	SE	Mean	SE		Mean	SE	Mean	SE	
**Growth performance**										
BW	293.66	3.15	250.92	3.23	<0.001	272.33	3.02	230.26	2.92	<0.001
WH	117.18	0.37	112.74	0.37	<0.001	113.84	0.32	109.31	0.39	<0.001
CG	153.52	0.62	147.54	0.65	<0.001	150.41	0.59	144.80	0.55	<0.001
**Blood metabolites**										
VitA	88.11	3.10	90.77	3.14	n.s.	89.13	3.82	89.72	3.81	n.s.
VitE	147.70	8.33	144.50	8.61	n.s.	172.46	11.12	165.07	10.05	n.s.
bC	93.51	6.20	95.39	6.17	n.s.	108.50	8.22	88.46	5.44	<0.05
AST	68.32	1.86	71.76	2.11	n.s.	72.72	2.46	71.22	1.47	n.s.
GGT	19.07	0.41	21.35	0.79	<0.05	22.18	0.72	21.26	0.73	n.s.
TC	106.31	4.01	111.88	4.05	n.s.	122.63	5.44	121.26	4.75	n.s.
ALB	3.63	0.03	3.61	0.02	n.s.	3.66	0.03	3.65	0.02	n.s.
BUN	11.15	0.34	12.35	0.47	<0.05	12.98	0.50	12.14	0.48	n.s.
**CW**	577.91	2.88	425.67	2.00	<0.001	515.19	3.63	365.97	2.70	<0.001

**Table 4:** Comparisons of growth performance, blood metabolites, and carcass weight between high and low body weight (BW) groups. Growth performance and blood metabolites were measured at the start of fattening (approximately 8–10 months). SE: Standard error. Abbreviations of traits are shown in Table 1.

Traits	Steer					Heifer				
	High (N = 57)		Low (N = 55)		*p*-Value	High (N = 43)		Low (N = 46)		*p*-Value
	Mean	SE	Mean	SE		Mean	SE	Mean	SE	
**Growth performance**										
BW	317.20	1.77	228.38	2.02	<0.001	286.01	2.15	209.61	1.46	<0.001
WH	118.11	0.37	111.12	0.35	<0.001	114.67	0.32	108.70	0.29	<0.001
CG	157.60	0.37	143.72	0.42	<0.001	152.84	0.48	140.62	0.55	<0.001
**Blood metabolites**										
VitA	85.35	2.97	91.06	3.40	n.s.	82.57	3.18	91.92	4.44	n.s.
VitE	163.88	9.98	126.64	5.58	<0.01	167.46	10.81	144.55	8.49	n.s.
bC	98.69	7.05	80.58	4.37	<0.05	116.85	9.63	85.59	6.46	<0.01
AST	67.45	2.15	74.37	2.74	<0.05	72.46	2.58	76.01	1.97	n.s.
GGT	19.25	0.50	20.41	0.87	n.s.	21.86	0.84	21.30	0.80	n.s.
TC	108.95	4.32	111.61	4.29	n.s.	125.45	6.06	112.64	6.12	n.s.
ALB	3.64	0.03	3.60	0.03	n.s.	3.74	0.04	3.65	0.03	<0.05
BUN	11.90	0.44	12.28	0.48	n.s.	11.87	0.50	12.49	0.57	n.s.
**CW**	538.77	6.88	458.85	5.03	<0.001	487.03	6.91	402.43	5.36	<0.001

## Data Availability

Not applicable.
